# Protoplast isolation and transient transformation system for *Ginkgo biloba* L.

**DOI:** 10.3389/fpls.2023.1145754

**Published:** 2023-03-15

**Authors:** Xin Han, Hao Rong, Yining Feng, Yue Xin, Xiaoyue Luan, Qi Zhou, Meng Xu, Li-an Xu

**Affiliations:** ^1^Key Laboratory of Forestry Genetics & Biotechnology of Ministry of Education, Co-Innovation Center for Sustainable Forestry in Southern China, Nanjing Forestry University, Nanjing, China; ^2^Zhejiang Academy of Forestry, Hangzhou, China

**Keywords:** *Ginkgo biloba* L., protoplasts, PEG-mediated transformation, transient expression, protein interaction

## Abstract

*Ginkgo biloba* L. has a unique evolutionary status. Owing to its high medicinal and ornamental value, ginkgo has also recently become a research hotspot. However, the large genome and long juvenile period, as well as the lack of an effective genetic transformation system, have hindered gaining a full understanding of the comprehensive functions of ginkgo genes. At present, heterologous expression of genes in model plants is the primary method used in ginkgo-related research; however, these distant plant model relatives limit reliable interpretation of the results for direct applications in ginkgo breeding. To overcome these limitations, in this study, an efficient isolation and transient expression system for ginkgo protoplasts was established. A large number of intact and homogeneous ginkgo mesophyll protoplasts were isolated using 2% cellulase and 0.25% pectinase in 0.4 M mannitol. The activity of these protoplasts remained above 90% even after 24 h. Furthermore, when the concentration of the polyethylene glycol 4000 solution was 30%–40% (w/v), the transformation efficiency of the protoplasts reached 40%. Finally, the reliability of the system was verified using subcellular localization, transient overexpression, and protein interaction experiments with ginkgo genes, thereby providing a technical platform for the identification and analysis of ginkgo gene functions. The proposed method partially compensates for the limitations associated with the lack of a genetic transformation system and provides technical support to expand research on elucidating the functions of ginkgo genes.

## 1 Introduction

*Ginkgo biloba* L. (commonly referred to as ginkgo or the maidenhair tree) is an ancient relict plant with a long history of cultivation and utilization in China, which led to its listing as a national key protected wild plant. Fossil evidence shows that ginkgo dates back to the Early Permian period (approximately 270 million years ago) and became widespread after the Quaternary glacial period. However, the morphological characteristics of ginkgo have not markedly changed over the past 100 million years (Zhou and Zheng, 2003). As an excellent landscaping tree, the most attractive ornamental feature of ginkgo is its distinctive leaf shape and golden yellow leaves in autumn (Li et al., 2018). As the only species of the Ginkgopsida family, ginkgo has a unique evolutionary position and has important research value for understanding the genetic evolution of plants. Moreover, because of its applications in the fields of medicine and horticulture, as well as its ornamental value, ginkgo has attracted broad research attention (Khalil et al., 2020). Guan et al. (2016) constructed the draft genome of ginkgo using second-generation sequencing technology. Recently, Liu et al. (2021) assembled a nearly complete high-quality genome of ginkgo. Although the whole-genome data of ginkgo (9.87 G) have been further improved with the development of sequencing technology, the structural and functional annotations of individual ginkgo genes remain largely unknown and require further in-depth study.

The slow progress of ginkgo gene research is mainly due to the unique characteristics of this species. First, the unique evolutionary status of ginkgo augments the difficulty of bioinformatics prediction. Second, despite many generations of research, there is still no effective stable genetic transformation system for ginkgo (Dupré et al., 2000; Zhang, 2003; Guo, 2004). To date, ginkgo gene research has mainly been carried out based on heterologous transformation, limiting the depth and accuracy of gene function studies (Wu et al., 2020; Xin et al., 2021). In addition, the ginkgo life cycle is long, its genetic background is complex, and the genetic transformation efficiency is low. At present, stable transformation cannot enable the rapid identification of related gene functions, understanding of unknown metabolic pathways, or artificial regulation (Li et al., 2020).

Transient gene expression based on plant protoplasts is a quick and efficient method, which can compensate for the limitations of other gene function research methods for species that have difficulties with stable genetic transformation. The transient transformation technique can be used to characterize the gene activity shortly after transformation (several hours to several days), and avoids the problem of the insertion position effect associated with stable transformation (Dekeyser et al., 1990). Using appropriate material selection and extraction methods, protoplasts can produce physiological responses similar to those in intact tissues and individual plants, as well as reproduce plant cellular regulatory processes such as subcellular localization, promoter activation, protein interactions, protein sorting, and vesicle transport (Kerppola, 2008; Faraco et al., 2011). Yoo et al. (2007) improved the protocol for leaf protoplasm extraction and transient transformation in *Arabidopsis thaliana*; this protocol has become the basis for establishing transient transformation systems in other species. Moreover, Tan et al. (2013) established a transient protoplast transformation system for perennial tree poplars. In recent years, protoplast isolation and transient transformation systems have been developed for other plants, such as rice (Chen et al., 2006; Poddar et al., 2020), maize (Sheen, 2001; Cao et al., 2014), cotton (Li et al., 2014), buckwheat (Sakamoto et al., 2020), tulip trees (Huo et al., 2017), and apple trees (Wang et al., 2021). Concurrently, with the development of technology, protoplast isolation systems for specific plant tissues such as maize endosperm (Hu et al., 2020) and orchid petals (Ren et al., 2021) have expanded the application prospects and development potential of transient transformation. However, transient transformation has thus far largely been applied to species under the angiosperm classification. To date, relevant studies on the protoplasts of ginkgo have only involved the preliminary isolation and purification of the mesophyll protoplasts (Lai et al., 2020), and the establishment and application of a transient transformation system for ginkgo protoplasts has not been discussed. A protoplast-based transient transformation system has gradually become an important research method in molecular biology because of its rapid and high-throughput characteristics. Therefore, it is important to construct an efficient protoplast isolation and transient transformation system for *Ginkgo biloba* L. to best explore the large amount of available genomic information of ginkgo (including transcriptome data) while overcoming the technological limitations of genetic manipulation of this unique species (Liu et al., 2021).

Here, we present a simple method for isolating the protoplasts of ginkgo seedlings and utilizing them for high-throughput transient gene expression. This method provides an effective experimental platform for the functional exploration and regulatory relationship verification of genes in ginkgo.

## 2 Materials and methods

### 2.1 Plant materials

**Figure 1:**
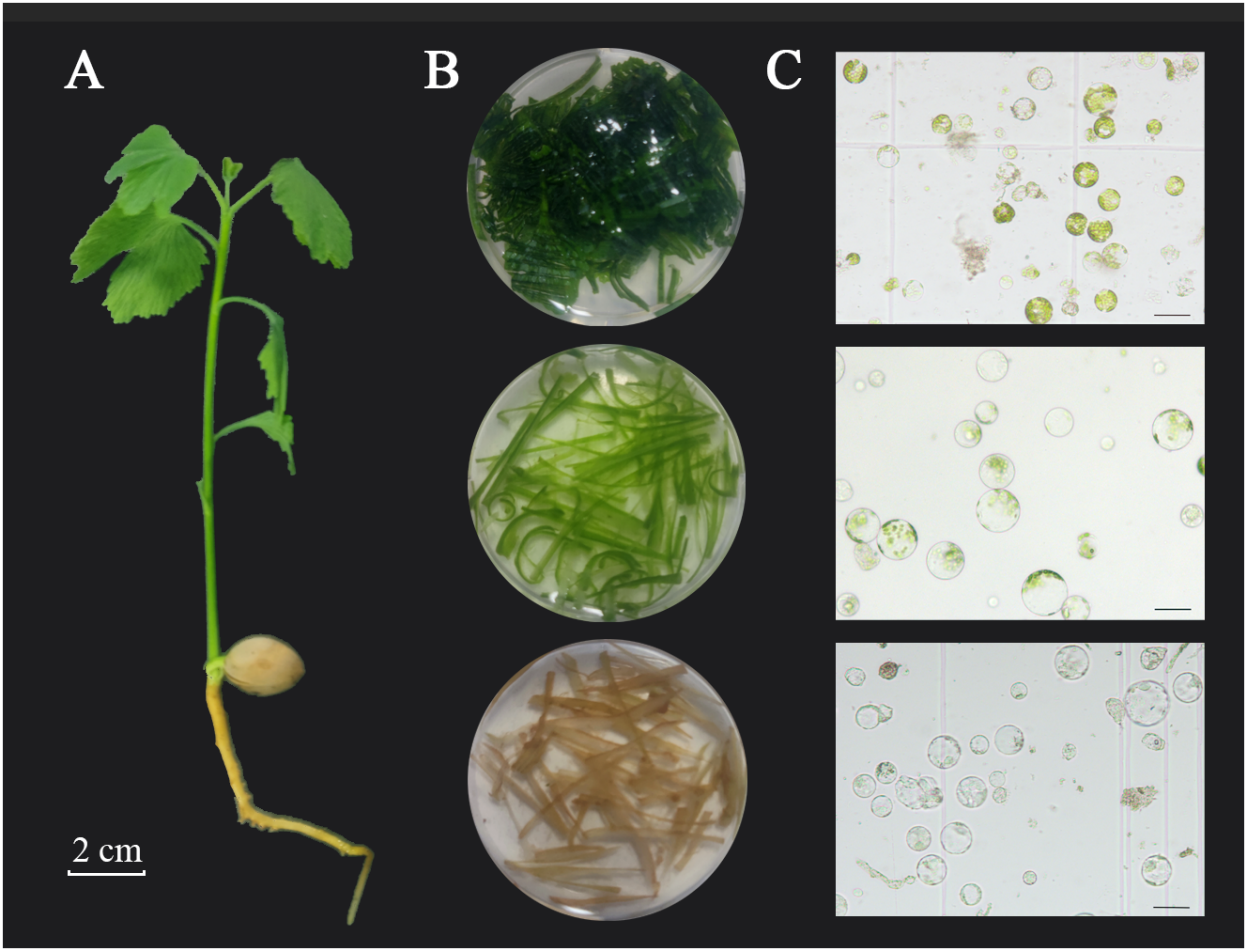
Isolation of protoplasts from different tissues of ginkgo seedlings. **(A)** Two-week-old ginkgo seedling; scale bar = 2 cm. **(B)** True leaves, tender stems, and young roots (from top to bottom) enzymatically hydrolyzed in enzymolysis solution. **(C)** Protoplast status in different tissues under bright-field microscopy; scale bars = 50 μm.

The *Ginkgo biloba* seeds used in this study originated from a mature female tree at the campus of Nanjing Forestry University (Nanjing, Jiangsu Province, China). The seeds were collected in September after the fruit was ripe and had fallen naturally, followed by sand accumulation treatment, and the seeds were stored at 4°C. After germination at 25°C, the seeds were sown in nutrient soil and placed under 15 h light:9 h dark, 65–80% humidity, and cultured in circulation at 25–28°C. After 2 weeks, the leaves, stems, roots, and other tissues from the ginkgo seedlings (**Figure 1**) were used for protoplast isolation and purification. In addition, the leaves from 30- and 90-day-old seedlings and adult ginkgo trees exhibiting healthy growth were collected for protoplast isolation. After obtaining all materials, the surfaces of the plant tissue were washed with soapy water, rinsed with sterile water five times, and left in water until used.

### 2.2 Vector construction

#### 2.2.1 Green fluorescent protein-tagged vector

The plasmid vector pJIT166 (4262 bp, **Supplementary Figure 1**) containing the CaMV 35S-GFP-NOS structure was used for transformation. This particular plasmid is a high-copy vector driven by a double 35S cauliflower-virus promoter (Huo et al., 2017). Ampicillin was used as the bacterial selection marker. To test whether the products of exogenous genes can be targeted *via* corresponding subcellular sites, we inserted the coding domain sequences (CDS) of *Chr3.406.1* and *Chr5.1249.1* upstream of *GFP*. PrimeSTAR^®^ Max DNA Polymerase (Code No. R045A, Takara Bio Inc., Japan) was used to perform *in vitro* amplification of the genes (primers are shown in **Supplementary Table S1**). After ligation into the pEASY^®^-Blunt Zero Cloning Vector (CB501, TransGen Biotech, Beijing, China), Trans1-T1 Phage Resistant Chemically Competent Cell (CD501, TransGen Biotech) was transformed for next-generation sequencing verification. The resulting gene sequence (with the stop codon removed) was linked to pJIT166-GFP *via* a double restriction site (KpnI-XhoI) to obtain *35S::Chr3.406.1-GFP* and *35S::Chr5.1249.1-GFP*. The ClonExpress^®^ II One-Step Cloning Kit (Code C112, Vazyme Biotech Co., China) was used for vector construction. Finally, the purified plasmids were analyzed and sequenced to confirm successful fusion construction.

#### 2.2.2 Overexpression vector

Similar to the method described above, the CDS of *Chr3.406.1* was linked to the pH35GS (11,739 bp **Supplementary Figure 1**) overexpression vector that had been modified in our laboratory to contain the CaMV 35S promoter and NOS terminator region. Spectinomycin was used as the bacterial selection marker. Sequencing was performed at each step to ensure the correct fusion.

#### 2.2.3 Bimolecular fluorescence complementation vector

As a negative control in the BiFC experiment, the no-load vector without the coding region of the target gene was combined with the vector obtained as described above. Simultaneously, the CDSs of the *Chr3.406.1* and *Chr5.1249.1* genes in *G. biloba* that may interact with each other were introduced into the NC-frame of the BiFC vector (Yan et al., 2020) without their termination codons (**Supplementary Figure 1**). *35S::Chr3.406.1/Chr5.1249.1-YFP^N^* and *35S::Chr3.406.1/Chr5.1249.1-YFP^C^* were also obtained, the primers for which are shown in **Table S1**. All plasmids were purified and then verified using sequencing.

### 2.3 Protoplast isolation

The optimal system for ginkgo protoplast isolation was determined by adjusting the key conditions according to the reported protoplast isolation methods for *Arabidopsis* and *Populus* (Yoo et al., 2007; Tan et al., 2013). After removing the ash and soil, the ginkgo tissues were collected and weighed, washed in sterile water, and the surfaces of the leaves were wiped before use. The stems, roots, and leaves from different tender degrees of ginkgo seedlings were selected (**Figure 1**). The tissues were cut into fine filaments (1.0 mm) with clean blades and then immediately placed into the prepared enzyme mixture for enzymatic hydrolysis. The enzyme-containing mixture included 20 mM KCl, 10 mM CaCl_2_, 0.1% bovine serum albumin, 0.2–0.6 M d-mannitol (DM), 20 mM 2-morpholinoethanesulfonic acid monohydrate (MES; pH 5.7), 2%–4% (v/v) cellulase (C2730; Sigma, St. Louis, MO, USA), and 0.25%–0.5% (v/v) pectinase (P2611; Sigma). DNAse and protease were inactivated by incubation at 55°C in a water bath for 10 min (Bai et al., 2020). The mixture was cooled to room temperature, filtered, and sterilized before use. After dark enzymatic hydrolysis at 28°C in standing or shaking (1×*g*) conditions for 3–6 h, an equal volume of W5 solution (2 mM MES, pH 5.7, 154 mM NaCl, 125 mM CaCl_2_, and 5 mM KCl) was added to stop the reaction. The undigested leaf tissue was screened using a 200 (75 μm) mesh stainless-steel cell filter, and the protoplasts were released into 50-mL round-bottom tubes. The protoplasts were then centrifuged for 5 min at 100 ×*g* with slight acceleration to form small spheres. The protoplasts were counted using a blood cell count board (#02270113; QIUJING^®^ Shanghai, China) under a microscope (×10 magnification). The protoplast number per gram of fresh tissue (gFW) was calculated as the protoplast yield. The prepared protoplasts were then placed on ice for 30–60 min until the cells settled at the bottom of the tube, after which the supernatant was removed. The protoplasts were resuspended in MMG solution (0.4 M mannitol, 15 mM MgCl_2_, 4 mM MES at pH 5.7) to a concentration of 6 × 10^5^ protoplasts per mL. After undergoing extraction for 24 h, the homogenized liquid was absorbed and 0.01% of fluorescein diacetic acid was added to monitor protoplast activity, which was calculated as (number of fluorescent protoplasts/total number of protoplasts) × 100%. The experiments were independently repeated at least three times.

### 2.4 Protoplast transformation

Transformation of ginkgo protoplasts was performed using a polyethylene glycol (PEG)–Ca^2+^ transformation method. The same batch of plasmid DNA and protoplasts was used for all experiments, and three replicates were prepared of the same group for each experiment. The process was repeated three times to reduce any errors caused by the batch effect. For transformation, 10 μg plasmid DNA and 5–6 × 10^4^ protoplasts (approximately 100 μL in volume) were used, and the procedure was optimized according to the two following key steps: PEG concentration and transform time. The above-mentioned mixture was gently mixed with an equal volume of PEG solution (0.2 M mannitol, 100 mM CaCl_2_) containing PEG 4000 (20%–50%, w/v). The mixture of protoplast, plasmid DNA, and PEG was transformed at 25°C, and the transformation time was set to 10, 20, 30, and 40 min. After transformation, 500 μL of W5 was mixed in to stop the reaction. Transformed protoplasts were collected by centrifuging at 100 ×*g* for 5 min at 25°C. After removing the supernatant, the protoplasts were resuspended in 100 μL WI solution (0.4 M DM, 4 mM MES at pH 5.7, 20 mM KCl) and then incubated in the dark for further analysis. A Zeiss Scope.A.1 fluorescence microscope (Carl Zeiss, Oberkochen, Germany) equipped with an AxioCam HRC (Carl Zeiss) was used for imaging. The microscope contained three filter sets with excitation (EX) and emission (EM) specific to Rhodamine B (Rhod; EX 545 nm, EM 620 nm), 4′, 6-diamidino-2-phenylindole (DAPI; EX 360 nm, EM 460 nm), and fluorescein isothiocyanate (FITC; EX 488 nm, EM 507 nm) and all fluorescence experiments were independently repeated at least three times. The transformation efficiency of a reaction was calculated by counting more than 10 fields, with a total cell count of approximately 100 protoplasts.

### 2.5 RNA extraction and RT-qPCR

The protoplasts used for RNA extraction were transformed using a 10-fold reaction system, incubated for 6–8 h, and centrifuged at 2500 ×*g* for 3–5 min. After removing the supernatant, the protoplasts were frozen in liquid nitrogen and stored at – 80°C until RNA extraction. Gene-specific primers were designed using Beacon Designer 8.14 (http://www.premierbiosoft.com/molecular_beacons/index.html) software. The ginkgo housekeeping gene *GbGAPDH* served as the reference gene (see **Supplementary Table 1** for primer information). PrimeScript™ RT Master Mix (Takara, Tokyo, Japan) was used for reverse transcription. T5 super mix (TsingKe, Beijing, China) was used for real-time PCR (RT-PCR), and the amplification was performed for 26 cycles at 98°C for 30 s, 60°C for 10 s, and 72°C for 10 s. PCR product was analyzed by agarose gel electrophoresis and staining with ethidium bromide. The reverse transcription product was diluted three-fold for real time quantitative polymerase chain reaction (RT-qPCR) performed using SYBR Premix Ex Taq (Takara, Tokyo, Japan) and a ViiA 7 Real-Time PCR System (Thermo Fisher, Waltham, MA, USA), with three replicates per sample reaction. PCR amplification was performed under the following conditions: initial denaturation at 95°C for 30 s, followed by 40 cycles of 95°C for 5 s, 60°C for 30 s, 72°C for 15 s, and then 95°C for 10 s. Relative expression levels were calculated using the 2^-ΔΔCt^ method.

### 2.6 Statistical analysis

All experimental data were analyzed using one-way analysis of variance and graphed using GraphPad Prism 8 (https://www.graphpad.com/). Multiple comparisons were performed using Tukey’s honest significant difference test, where *p* < 0.05 indicated a statistically significant difference.

## 3 Results

### 3.1 Selection of optimal materials for protoplast isolation of ginkgo

**Figure 2:**
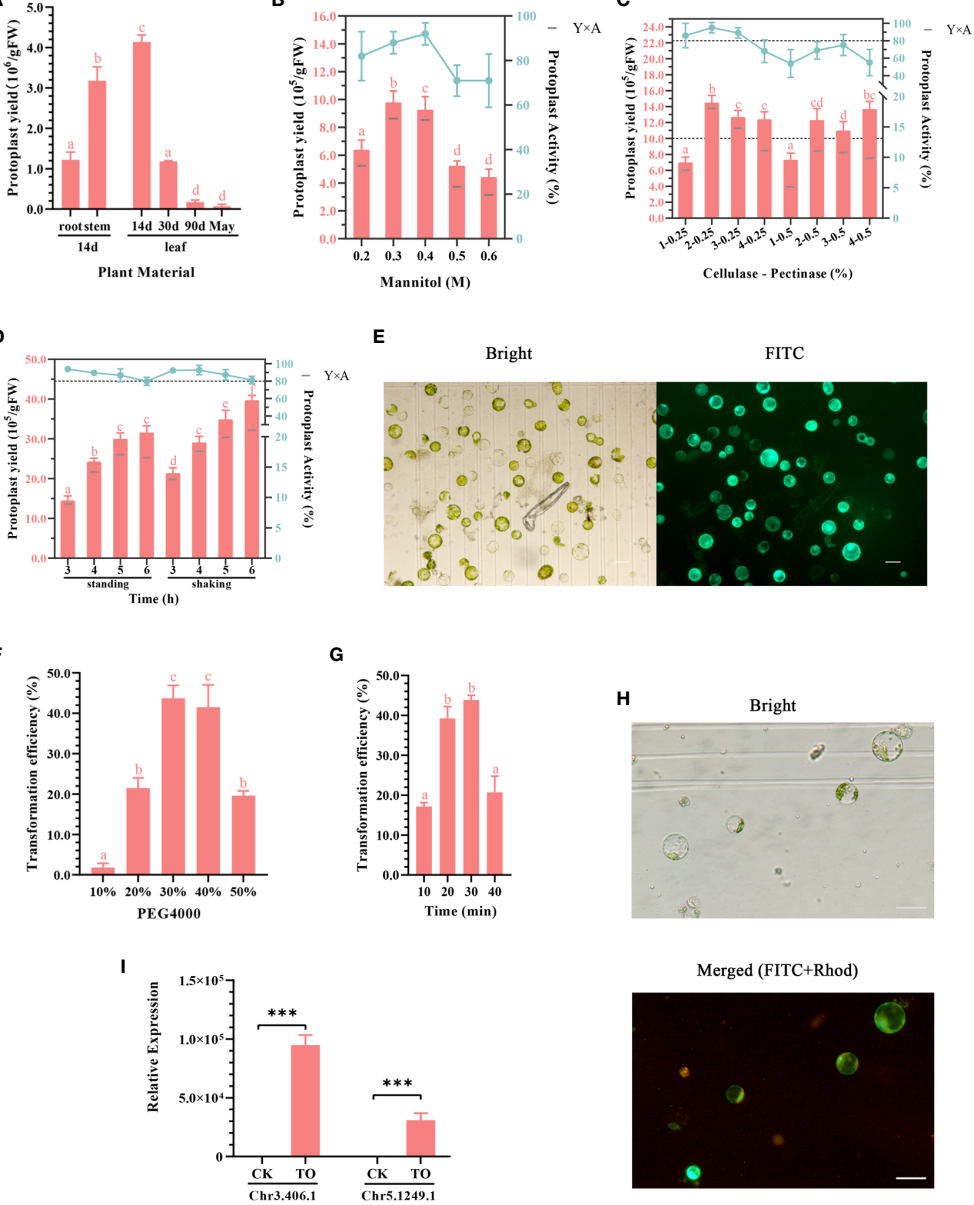
**(A–E)** Optimization of the ginkgo protoplast isolation system. **(A)** Effects of different plant materials on protoplast isolation efficiency. The enzymatic solution composition was 0.4 M mannitol, 2% (v/v) cellulose, and 0.5% (v/v) pectinase; the enzymatic hydrolysis conditions were constant temperature of dark enzymatic hydrolysis of 28°C for 3 h; the tissue mass in each group was approximately 0.5 g. **(B)** Effect of mannitol concentration on protoplast yield and activity. The enzymatic solution was consistent with that described in **(A)**. Approximately 0.3 g leaves from 2-week-old seedlings were subjected to enzymatic hydrolysis for 3 h. The bar chart shows protoplast production and the broken line shows protoplast activity in 24 h. Y×A is the product of the number of isolated protoplasts and their average activity, which was calculated as the number of active protoplasts in 24 h. **(C)** Effects of cellulase and pectinase contents on protoplast yield and activity. The mannitol concentration was 0.4 M; 0.3 g leaves from 2-week-old seedlings were subjected to enzymatic hydrolysis for 3 h. The two dotted lines indicate the protoplast yield of 10.0 × 10^5^/gFW and activity of 80%, respectively. **(D)** Effect of enzymatic hydrolysis time on protoplast yield and activity. Enzymatic solution composition: 0.4 M mannitol, 2% (v/v) cellulose, and 0.25% (v/v) pectinase. Enzymatic hydrolysis conditions: allowed to stand or shaken at 1 ×*g* at 28°C for 3–6 (h) Different lowercase letters above bars in **(A–D)** indicate a significant difference (*p* < 0.05). **(E)** Fluorescein diacetate (FDA) staining was used to detect the activity of ginkgo protoplasts after 24 h; scale bar = 50 μm. The left image shows the bright field and the right image shows the FITC channel. Green light represents the active protoplasts. **(F–H)** Optimizing the conditions of the ginkgo protoplast transient transformation system. **(F)** Effects of different concentrations of PEG 4000 on transformation efficiency. Conversion solution composition: 10 μg plasmid, approximately 5 × 10^4^ ginkgo protoplasts, and 10%–50% PEG 4000 solution. Transformation conditions: allowed to stand at 25°C for 30 min. **(G)** Influence of transformation time on transformation efficiency. The transformation solution was 30% (w/v) PEG 4000 and the same components described above for **(F)**. Transformation conditions: allowed to stand at 25°C for 10–40 min. Different lowercase letters above bars in **(F–G)** indicate a significant difference (*p* < 0.05). **(H)** *35S::GFP* transformation of ginkgo protoplasts confirmed under a fluorescence microscope at 10× magnification; scale bar = 50 μm. The upper image shows the bright field and the lower image shows the merge of the FITC and Rhod channels. Red light represents autofluorescence and green light represents the protoplasts successfully transformed. **(I)** RT-qPCR results for the *Chr3.406.1* and *Ghr5.1249.1* genes that were transiently overexpressed in ginkgo protoplasts (TO); ***p < 0.001.

To obtain high-quality ginkgo protoplasts as quickly as possible, four types of tissues, including the roots, stems, and leaves of ginkgo seedlings as well as mature ginkgo leaves, were used to explore the influence of plant tissue status on the protoplast isolation efficiency (**Figure 1**). The results showed that the degree of leaf development had a significant impact on the protoplast isolation efficiency (**Figure 2A**), which gradually decreased with leaf development. Specifically, there was minimal difference in the number of protoplasts isolated from the leaves of 90-day-old seedlings and adult trees. Given the limited biomass of 2-week-old seedlings, we also compared the isolation efficiency and status of protoplasts from different ginkgo tissues. As shown in **Figure 1C**, the root cells of ginkgo did not contain chloroplasts, and the cell structure under the visual field was blurred. Compared with cells from the mesophyll, the sizes of cells in the roots and stems varied greatly. In terms of isolation efficiency, the protoplast yield of the leaves and stems from 2-week-old seedlings exceeded 3 × 10^6^/gFW, characterizing these tissues as ideal extraction materials. Considering that the establishment of a transient transformation system requires uniform cells, we selected the leaves of 2-week-old ginkgo seedlings as the material for protoplast isolation in the subsequent experiments.

### 3.2 Optimization of protoplasmic isolation from the true leaves

Mannitol is a common osmotic pressure regulator and plays an important role in the maintenance of plant protoplasts in the *in vitro* culture environment. Generally, the optimal mannitol concentration that maintains the osmotic pressure in plant cells ranges from 0.4 to 0.6 M (Xu and Wei, 1997). Appropriate osmotic pressure is important for improving the reliability of subsequent experiments and the long-term activity of protoplasts. Compared with *Arabidopsis* and *Populus*, ginkgo harbors cells with larger differences in cell size. Therefore, we set the concentration of mannitol to 0.2–0.6 M for the gradient experiment (**Figure 2B**). Under the condition of 0.3 M mannitol, the protoplast yield was the highest, whereas the mannitol activity was the highest with a concentration of 0.4 M. Therefore, we calculated the performance under both concentrations and compared the yield of 24-h active protoplasts. The results showed that both the 0.3 and 0.4 M mannitol systems could obtain more active protoplasts from the ginkgo mesophyll, and the cell activity in the 0.4 M mannitol environment remained high (up to 90%) after 24 h.

Plant protoplasts are normal plant cells that do not contain cell walls. The common enzymes involved in the process of enzymatic hydrolysis include cellulase, pectinase, and macerase. Based on the results of the preliminary experiment, the more convenient and efficient aqueous enzyme solution containing cellulase and pectinase was used as the enzyme source in this study. The experimental group included varying amounts of cellulase and pectinase and was subjected to varying durations of enzymatic hydrolysis. The protoplast yield and protoplast activity after 24 h were measured and multiplied to obtain the number of active protoplasts (**Figure 2C**). The results showed that the mixture containing 2% cellulase and 0.25% pectinase produced relatively large protoplasts at 1.4 × 10^6^ protoplasts/gFW after reaction for 3 h. According to the statistics on the yield of protoplasts with activity after 24 h (Y×A), it was found that 2%–3% cellulase had a good effect, with more than 1 × 10^6^ protoplasts/gFW exhibiting activity.

To explore the optimal enzyme ratio, we found that the protoplasts isolated during the enzymatic process had obvious sedimentation and aggregation within the six-well plate. Therefore, the treatment conditions for enzymatic hydrolysis were also tested (**Figure 2D**). The results showed that the protoplast yield was higher under gentle shaking at 1 ×*g* as opposed to standing still for the same treatment time. In general, cell activity decreased slightly with increasing reaction time. Under the dark condition, the number of protoplasts did not increase significantly after enzymolysis for 5 h; however, the activity of the cells decreased. This may be related to the toxic effect of enzymes on the cells. Taken together, this optimization experiment showed that high-quality protoplasts (**Figure 2E**) could be isolated by performing enzymolysis at 28°C under dark conditions with shaking at 1 ×*g* for more than 3 h.

### 3.3 Optimization of the transient transformation system of ginkgo protoplasts

For the transient transformation of protoplasts mediated by PEG, the number of protoplasts, concentration of plasmids, concentration of PEG, and transformation time were the main factors affecting the efficiency. Based on the results of the preliminary experiment, we found that the concentration of PEG 4000 and the transformation time were particularly important for maximizing the transient transformation efficiency of ginkgo protoplasts under sufficient plasmid and protoplast conditions. Appropriate parameters must be determined for both transformation efficiency and cell activity. As shown in **Figures 2F–H** the protoplasts could effectively absorb foreign plasmid DNA when the PEG 4000 concentration exceeded 20% and the transformation time was set to 30 min. When using a 50% concentration of PEG 4000, the protoplasts of ginkgo were obviously damaged and the transformation efficiency was decreased. Therefore, 30%–40% (w/v) was determined to be the suitable PEG 4000 concentration for the transient transformation of ginkgo mesophyll protoplasts.

For the transformation time at the same PEG concentration, the transformation efficiency increased with increasing time; however, there was no significant improvement after 20 min (approximately 40%). When the reaction time reached 40 min, the protoplasts were damaged and the transformation efficiency was attenuated. Because PEG can increase the permeability of cell membranes, fragile protoplasts can easily be destroyed (Li et al., 2020). Therefore, increasing the transformation efficiency in a short time helps to maintain cell activity. These results suggested that 30%–40% (w/v) PEG 4000 and approximately 20 min for the mixing reaction as the most suitable conditions for the transient transformation of ginkgo protoplasts.

### 3.4 Application of the transient transformation system

The establishment of a protoplast isolation and transient transformation system for ginkgo provides a platform for downstream experiments. The transient transformation efficiencies of mesophyll protoplasts in *Arabidopsis* and *Populus* are close to 90% and 80%, respectively (Yoo et al., 2007; Tan et al., 2013) Conversely, the current transient transformation efficiency of ginkgo is only 40%. To this end, we carried out further experiments, including subcellular localization, transient overexpression, and BiFC, to verify the effectiveness of the system in practical settings.

#### 3.4.1 Subcellular localization

**Figure 3:**
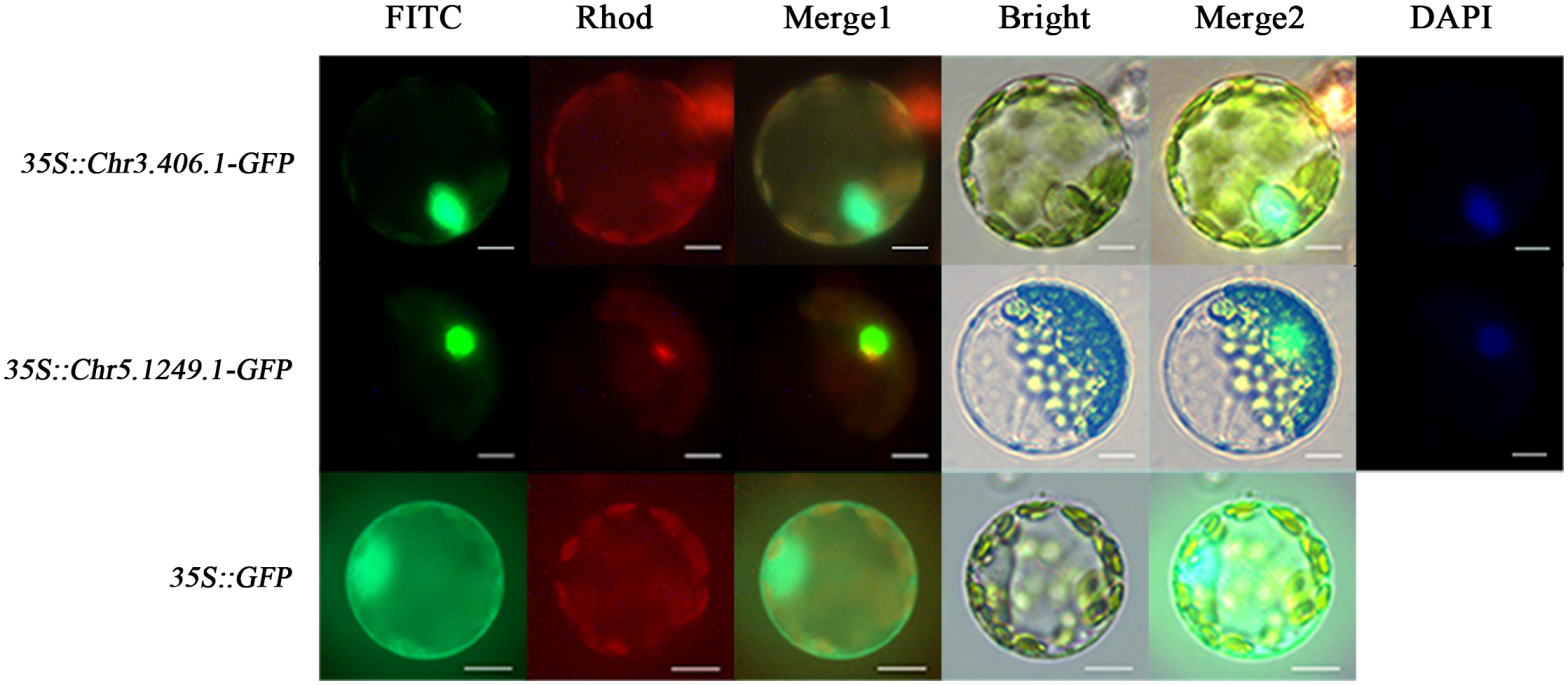
Subcellular localization for different vectors in ginkgo protoplasts (scale bars = 10 μm). The protoplasts transformed by three vectors are displayed in different channels. The FITC channel shows the location of the GFP fusion gene, the Rhod channel shows the autofluorescence of protoplasts, and the Bright channel shows the cell state. Merge1 is the combination of FITC and Rhod channels and Merge2 is the integration of FITC, Rhod, and Bright channels. DAPI is the nuclear marker.

To test whether the products of exogenously imported genes can be targeted to corresponding subcellular sites in the ginkgo protoplasts, we transformed the protoplasts with *35S::Chr3.406.1-GFP*, *35S::Chr5.1249.1-GFP*, and *35S::GFP* (pJIT166), respectively (**Figure 3**). Owing to the lack of a localization signal, GFP fluorescence of the empty carrier was uniformly dispersed throughout the protoplast. GFP fluorescence associated with transformation of the transcription factors *Chr3.406.1* and *Chr5.1249.1* was restricted to the nucleus, as shown by its colocalization with DAPI (a nuclear localization dye; **Figure 3**).

#### 3.4.2 Transient overexpression

Preliminary transcriptome data and quantitative results (Han et al., 2021) indicated that *Chr3.406.1* and *Chr5.1249.1* are seed-specific B3 transcription factors that exhibit almost no expression in the leaves of ginkgo. In this study, we used an expression vector constructed with the CaMV 35S promoter to transform the ginkgo protoplasts transiently. After incubation for 6 h with untransformed protoplasts serving as the controls, the RNA was extracted for RT-qPCR. As shown in **Figure 2I** (raw data in **Supplementary Table 2**), the RNA expression levels in the transformed protoplasts were significantly higher than those in the control protoplasts. This was also confirmed by RT-PCR results in **Supplementary Figure 2**. Therefore, with our proposed method, ginkgo genes appear to be normally expressed in the protoplasmic cells of the original plant, as driven by the CaMV 35S promoter.

#### 3.4.3 Protein–protein interaction analysis using the BiFC assay

**Figure 4:**
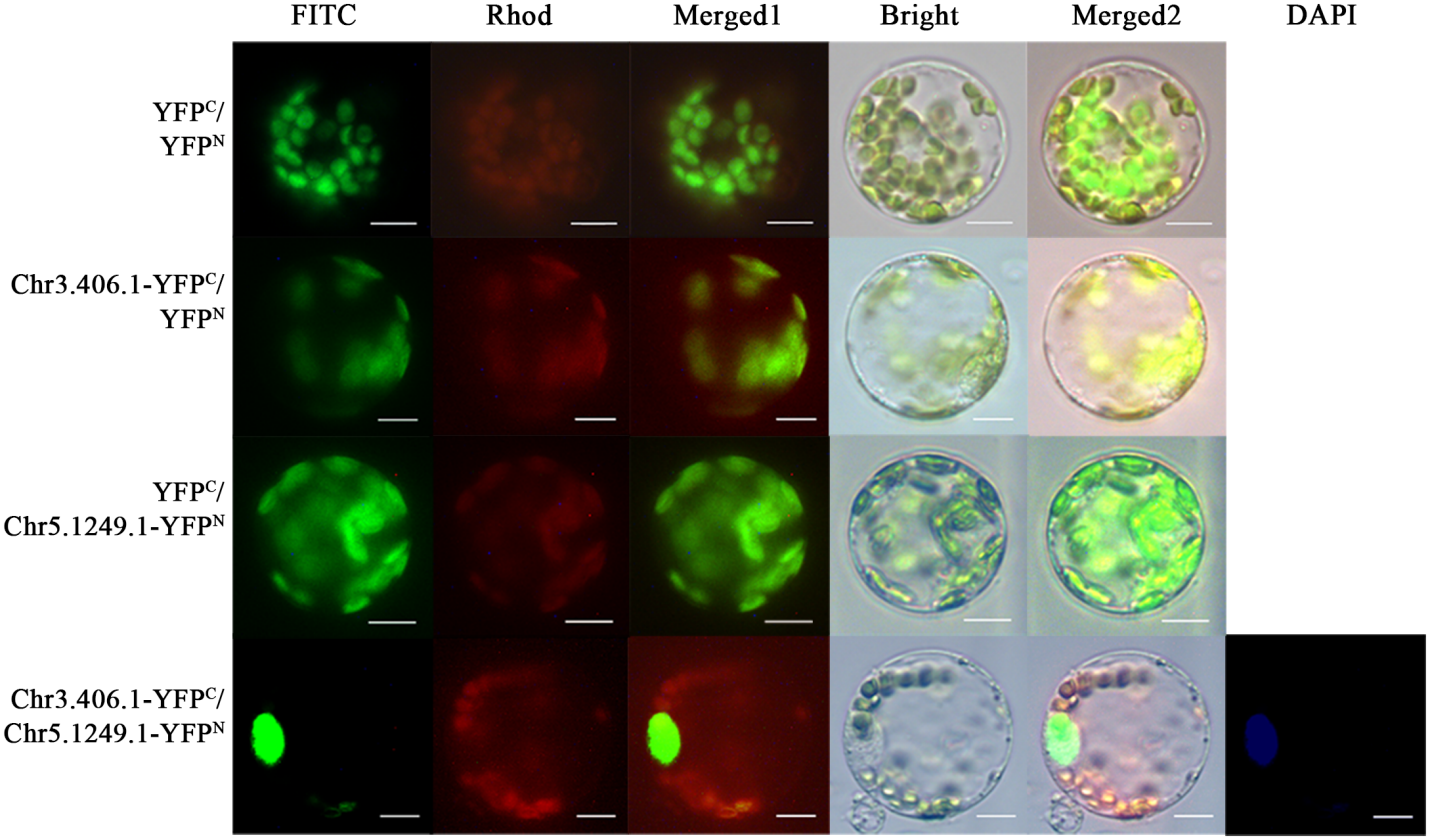
Protein–protein interaction assays in ginkgo protoplasts. Constructed pairs of Chr3.406.1-YFP^C^ with Chr5.1249.1-YFP^N^ (predicted), Chr3.406.1-YFP^C^ with YFP^N^ (negative control), YFP^C^ with Chr5.1249.1-YFP^N^ (negative control), and YFP^N^ with YFP^C^ (negative control) were transiently transformed in ginkgo protoplasts. The BiFC fluorescence is indicated by the YFP signal (FITC channel, channel imaging shown in green). Individual and merged images of YFP and chlorophyll autofluorescence as well as bright-field images of protoplasts are shown. DAPI is the nuclear marker. Scale bars = 20 μm.

In the full-length transcript dataset of ginkgo seed development (Han et al., 2021), we selected a pair of potentially interacting protein-coding genes, *Chr3.406.1* and *Chr5.1249.1*, which have been reported as key transcription factors in the regulation network of the seed plants. We fused the N- and C-termini of yellow fluorescent protein (YFP) and *Chr3.406.1* and *Chr5.1249.1* to obtain pairwise co-transformation vector combinations, pNC-Chr3.406.1-YFP^N/C^ and pNC-Chr5.1249.1-YFP^N/C^, respectively. The group of fusion genes was transiently co-transformed in the protoplasm of ginkgo and the yellow fluorescence signal was recovered in the protoplast (**Figure 4**). The negative control did not exhibit any YFP signals.

## 4 Discussion

*Ginkgo biloba* L. is an ancient gymnosperm that is the only surviving species in the Ginkgopsida family since the Quaternary glacial period. In phylogenetic analyses, the ginkgo taxon belongs to one of the five existing seed plants (Donoghue and Doyle, 2000) and is a sister group to the cycads (Liu et al., 2022). In addition, recovered plant fossils support the hypothesis that ginkgo originated from seed ferns (Herrera et al., 2017), indicating the importance of ginkgo in the phylogeny of terrestrial plants. Many omics studies have been conducted to better understand this species; however, the extensive amount of genetic information generated through these efforts has not yet been deeply explored and verified, mainly because a transformation system for this species has not been developed. Although many researchers have carried out studies on callus culture (Zhang, 2003) and embryoid induction (Guo, 2004) in ginkgo, there has still been no effective transformation technology developed for the functional verification of ginkgo genes. As a result, the current genetic research on ginkgo relies on the transformation of heterologous plants (Su et al., 2020; Xin et al., 2021), thereby posing many limitations in interpretation and practice due to the unreliability of distant kin. This study is the first to establish an isolation and transient transformation system using ginkgo protoplasts, offering important application value that could provide technical support for future study of the function and regulation processes of ginkgo genes.

Currently, the plant materials frequently used to isolate plant protoplasts mainly include cotyledons, tissue culture seedlings, and the young leaves of healthy seedlings (Huo et al., 2017; Bai et al., 2020; Gou et al., 2020). Our experiments with ginkgo indicated that only the leaves and stems from the seedlings could be used as relatively stable isolation and transformation materials. However, even for leaves among the seedlings, the extraction efficiency of protoplasts differed significantly among different developmental states (**Figure 2A**). Notably, ginkgo seeds require sand storage treatment to break the dormancy from harvest to germination and lose their germination ability in storage conditions (Feng et al., 2018). Therefore, the isolation conditions for *G. biloba* protoplast materials are more stringent than those for other plants with established transient protoplast transformation systems. The optimal concentration of 0.4 M mannitol determined in this study is similar to that used for *Arabidopsis thaliana* (Yoo et al., 2007), but is lower than that used for *Populus* (Tan et al., 2013). This may be related to the different membrane protein characteristics and inclusions between the different plant species (Zonia and Munnik, 2007). In this experiment, we found that the cell integrity decreased significantly following an increase in mannitol concentration (**Figure 2B**). We also tested 0.8 M of mannitol for the enzymatic hydrolysis of ginkgo protoplasts and found that only a small amount of protoplasts were obtained, as previously reported (Lai et al., 2020); moreover, these protoplasts had poor stability, with their activity decreasing significantly after 24 h. This may be related to the source and treatment of the materials (Choury et al., 2018). Common enzymes in enzymolysis solutions include cellulase, hemicellulase, pectinase, macerozyme, and driselase (Ren et al., 2021). These enzymes can decompose cellulose, pectin, and other substances in the plant cell walls to release protoplasts. Owing to the different biological sources of these enzymes, they may have different optimal pH and temperature values. Based on a previous study on the protoplast isolation system of *Populus* conducted in our laboratory (Tan et al., 2013) and the preliminary experimental results of this study, we characterized the efficacy of cellulase and pectinase in the enzymolysis solutions. Compared with other species (Ren et al., 2021), a high cellulase content significantly increased the yield of ginkgo mesophyll protoplasts (**Figure 2C**).

The reaction conditions during enzymatic hydrolysis were similar to those reported for other species (Yoo et al., 2007) and shaking at low velocity was also conducive to protoplast isolation. A previous study also showed that low-speed centrifugation during enzymatic hydrolysis can reduce protoplast damage and help in maintaining cell activity (Yu et al., 2017). The PEG-mediated method is a relatively simple, low-cost, and efficient method for the transient transformation of protoplasts (Cao et al., 2014). Transient transformation of ginkgo showed that 30%–40% (w/v) PEG 4000 could effectively mediate foreign plasmid entry into protoplasts. When the PEG concentration was increased to 50% (w/v), the predicted transformation events were also high. However, in the actual observation, we found that 50% (w/v) PEG 4000 seriously affected protoplast activity. Cracked cells and cell contents scattered in the environment were visible under a microscope, which is not conducive to subsequent observation and quantification. PEG was originally used as a cell-fusion agent in protoplast fusion studies (Kao and Michayluk, 1974). PEG has the function of binding cells and disturbing the structure of the binding membrane phospholipid bilayer, which will cause some degree of damage to protoplasts (Xu and Wei, 1997). Therefore, 30%–40% (w/v) PEG 4000 treatment for 20 min was considered to be the best reaction condition. Nevertheless, the transient transformation efficiency of ginkgo protoplasts remained below 50%, which may be related to the characteristics of ginkgo mesophyll cells and the quality of protoplasts and plasmids. In addition, when the protoplasts of ginkgo were transformed 24 h after isolation, the transformation efficiency decreased (which was closely related to the stability of protoplast activity). We also found differences in the transformation efficiencies among protoplasts obtained from the leaves of seedlings in different seasons, which may be related to external factors such as ambient temperature. Taken together, the interaction of various factors resulted in low efficiency and poor stability of the protoplast transient transformation for ginkgo, thereby reflecting the difficulty in establishing such a system.

We qualitatively and quantitatively evaluated the application value of this system. Considering the slow progress in the study of gene function in ginkgo to date, three candidate proteins were selected for verification. The experimental results showed that different localization signals were correctly recognized (**Figure 3**). On this basis, large-scale subcellular localization of ginkgo genes was carried out in our laboratory and accurate results were obtained (Han et al., 2022). BiFC is a commonly used qualitative experimental method for protoplasts (Kerppola, 2008). In this study, the ginkgo interacting proteins were verified and the feasibility of the BiFC experiment in ginkgo protoplasts was confirmed. This experiment indicated that the quality and conversion efficiency of the ginkgo protoplasts were sufficient to characterize protein interactions and further demonstrated the application potential of the system.

The application of transient gene overexpression technology is particularly important for ginkgo, which lacks a stable genetic transformation system to promote related research on ginkgo gene function. In this study, we transiently overexpressed ginkgo genes in protoplasts and performed quantitative verification at the RNA level, reflecting the applicability of this system in transient overexpression and RNA interference experiments. Given that protoplasts contain the complete genome, it can be predicted that this system will also play a role in transcriptional regulation and further downstream experiments. Even with the modified system described in this study, the efficiency of transient transformation of ginkgo protoplasts is still low compared to that of most plants, highlighting the need for further improvement. In addition, the strict requirements for obtaining plant materials and the influence of protoplast activity on the transformation further reflect the difficulty of transient transformation in ginkgo; elucidating the precise reasons for such limitations necessitates further studies.

## 5 Conclusion

This study established a simple, efficient, and reliable system for the isolation and transient expression of protoplasts from ginkgo leaves. The system included protoplast isolation from seedlings, PEG-mediated transient DNA transformation, and downstream experimental analyses. The optimized transformation efficiency was found to be 40%. With this system, protoplast isolation and transient transformation of ginkgo can be completed in approximately 5 hours, and the experimental results can be obtained within 3 days (according to different experimental purposes). This system can be used for common downstream qualitative and quantitative experiments such as protein subcellular localization, transient gene expression, and protein interaction analysis. Ultimately, this system provides a stable and reliable platform for the transient transformation and analysis of ginkgo genes.

## Data availability statement

The original contributions presented in the study are included in the article/**Supplementary Material**. Further inquiries can be directed to the corresponding authors.

## Author contributions

LX, MX, and XH conceived and designed the project. XH, YF, and XL undertook the molecular biology experiments. XH, HR, and YX participated in the data analysis. XH drafted the manuscript. LX, HR, QZ, and MX modified the manuscript. All authors contributed to the article and approved the submitted version.

## Supplementary material

Supplementary Figure 1 Vector maps.

Supplementary Figure 2 RT-PCR of transient overexpression.
