# Supplementary figures and images for: Protoplast isolation and transient transformation system for Ginkgo biloba L
Source: Front Plant Sci. 2023 Mar 15;14:1145754. doi: 10.3389/fpls.2023.1145754 (PMC10099357; doi:10.3389/fpls.2023.1145754)

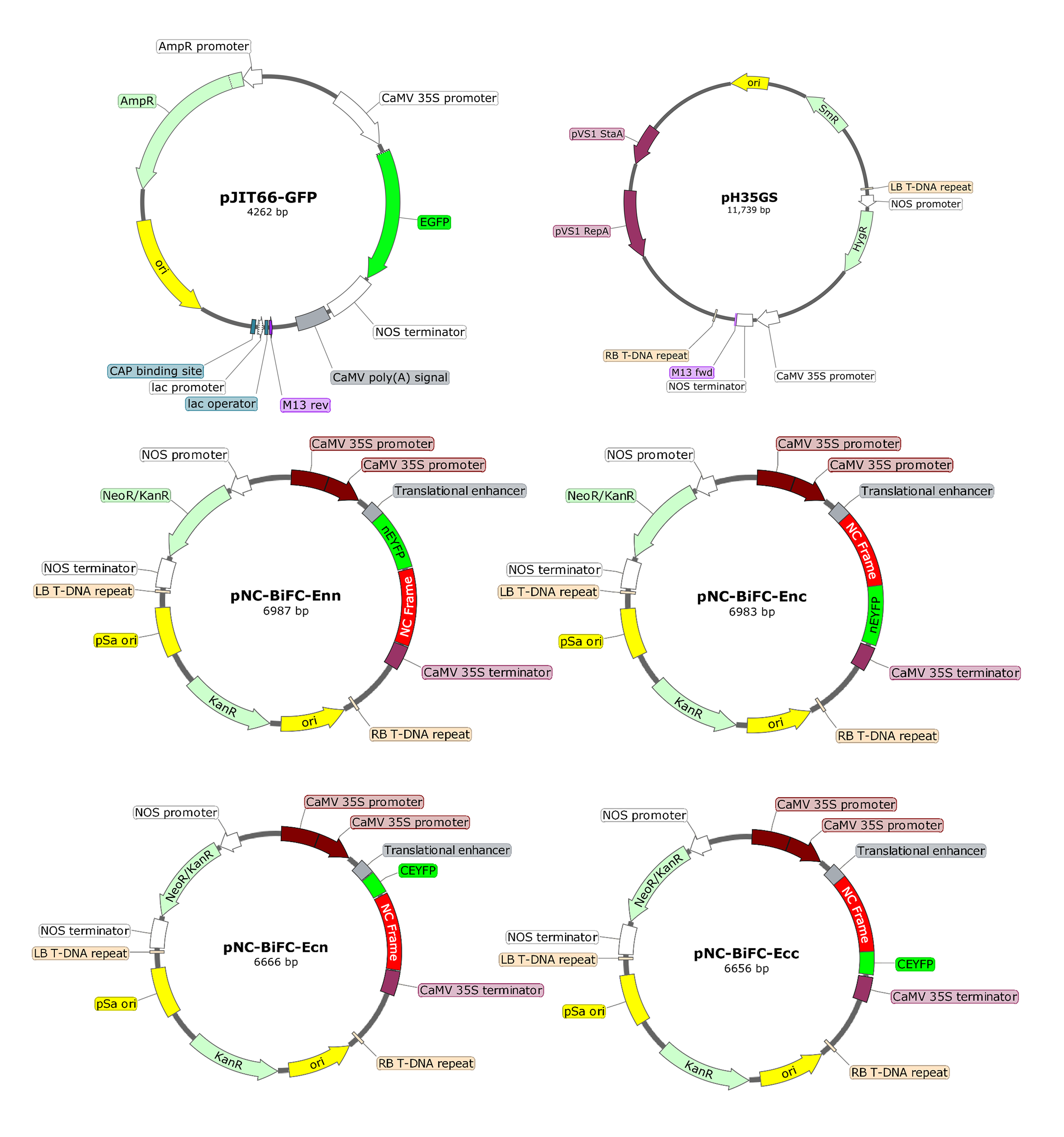

Supplement: Supplementary Figure 1 — Vector maps. [file Image_1.tif]

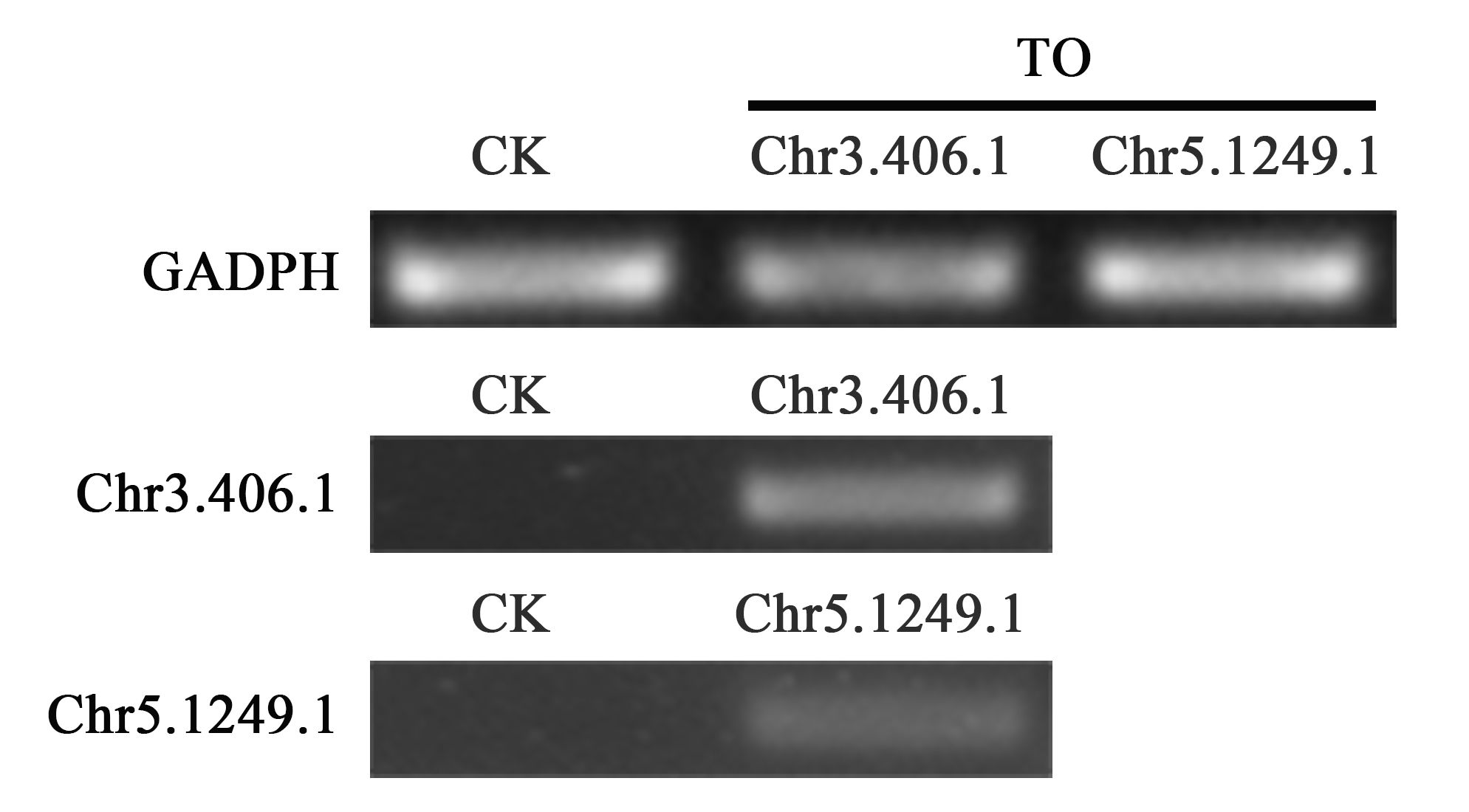

Supplement: Supplementary Figure 2 — RT-PCR of transient overexpression. [file Image_2.tif]
